# Impact of Chemotherapy Alone and in Combination with Immunotherapy on Oral Microbiota in Cancer Patients—A Pilot Study

**DOI:** 10.3390/microorganisms13071565

**Published:** 2025-07-03

**Authors:** Adriana Padure, Ioana Cristina Talpos-Niculescu, Paula Diana Ciordas, Mirabela Romanescu, Aimee Rodica Chis, Laura-Cristina Rusu, Ioan Ovidiu Sirbu

**Affiliations:** 1 Multidisciplinary Center for Research, Evaluation, Diagnosis and Therapies in Oral Medicine, “Victor Babes” University of Medicine and Pharmacy, 300041 Timisoara, Romania; adriana.padure@umft.ro (A.P.); laura.rusu@umft.ro (L.-C.R.); 2 Clinic of Oro-Dental Diagnosis and Ergonomics, “Victor Babes” University of Medicine and Pharmacy, 300041 Timisoara, Romania; ioana.talpos-niculescu@umft.ro; 3 Doctoral School, “Victor Babes” University of Medicine and Pharmacy, 300041 Timisoara, Romania; paula.muntean@umft.ro (P.D.C.); mirabela.romanescu@umft.ro (M.R.); 4 Biochemistry Discipline, “Victor Babes” University of Medicine and Pharmacy, 300041 Timisoara, Romania; ovidiu.sirbu@umft.ro; 5 Center for Complex Network Science, “Victor Babes” University of Medicine and Pharmacy, 300041 Timisoara, Romania; 6 Clinic of Oral Pathology, “Victor Babes” University of Medicine and Pharmacy, 300041 Timisoara, Romania

**Keywords:** microbiota, neoplasm, antineoplastic agents, immunotherapy, DNA sequencing

## Abstract

The oral cavity harbors a highly intricate and dynamic microbial ecosystem of multiple microhabitats supporting diverse microbial populations. As the second most complex microbiome in the human body, surpassed only by the gut, the oral microbiome comprises over 1000 species. Disruptions in the microbial balance have been associated with an increased risk of both oral diseases (dental caries and periodontitis) and systemic conditions, including inflammatory diseases and certain types of cancers. In our pilot study, we purified bacterial DNA from pre-treated, saponin-based, host-depleted saliva samples and performed 16S amplicon sequencing, using Oxford Nanopore Technologies, to identify bacterial composition and investigate changes in the oral microbiota of patients with solid tumors in response to chemotherapy, either alone or in combination with immunotherapy. We found significant reductions in microbial diversity of the oral microbiota following cancer treatment, which may contribute to post-therapeutic complications such as oral mucositis. Moreover, our findings indicate that on the one hand, following chemotherapy treatment the microbial profile is characterized by an increased abundance of *Streptococcus*, *Gemella*, and *Granulicatella* and a decrease in the abundance of *Neisseria* and *Veillonella*. On the other hand, post combined treatment, only *Streptococcus* relative abundance increased, *Veillonella* exhibited a slight decline, and *Haemophilus* and *Neisseria* displayed a marked decrease, whilst *Granulicatella* and *Gemella* remained relatively stable. Our findings underline the impact of cancer therapy on the oral microbiome, highlighting the potential for precision-based strategies to restore microbial balance and minimize treatment-related complications.

## 1. Introduction

The oral cavity hosts a highly dynamic and diverse microbial ecosystem of multiple microhabitats supporting intricate microbial communities, second in complexity only to the gut [1,2,3]. In a state of eubiosis, the microbial community coexists symbiotically with the host, contributing to immune modulation, maintaining mucosal barrier integrity, and inhibiting pathogenic colonization [4]. However, this balance is susceptible to disruption by numerous factors, including poor oral hygiene, antibiotic exposure, diet, systemic illnesses, and medical treatments such as chemotherapy [5,6]. Dysbiosis, defined as a shift toward a less diverse, pathogen-dominated microbial community, has been implicated in both oral and systemic diseases, highlighting a bidirectional relationship between the oral microbiome and host health.

Poor oral hygiene can lead to the development of dental plaque, which can stimulate the proliferation of bacteria (such as *Porphyromonas gingivalis*, *Bacteroides forsythus*, and *Treponema denticola*) producing enzymes and metabolites that could contribute both to local inflammation [7] and systemic inflammation [8]. Moreover, immune response [9] and drug-related side effects [10] were reported to be influenced by oral hygiene. Dietary habits such as high carbohydrates intake can lead to a proliferation of bacteria that produce acids, with a reduction in salivary pH which further induces the development of aciduric and acidogenic species, including *Streptococcus mutans* and *Lactobacillus species*, contributing to dysbiosis and dental caries [5,11]. Malnutrition can disrupt oral homeostasis, thereby contributing to disease progression within the oral cavity, by reducing the host’s resistance to microbial biofilm formation and diminishing the tissue’s ability to heal effectively [12]. Antibiotic use has been shown to reduce commensal *Actinobacteria* levels [7], while systemic illnesses like diabetes and immunosuppressive conditions (e.g., transplantation, or autoimmune conditions) can further disrupt host–microbiome interactions, favoring opportunistic pathogens such as *Klebsiella* and *Acinetobacter* [13,14].

These alterations in oral microbiome homeostasis are increasingly recognized as contributors not only to local diseases, such as dental caries, periodontitis, and oral cancer [5,15,16], but also to systemic conditions [5], including cardiovascular diseases, rheumatoid arthritis, visual impairment [17], bacterial pneumonia [18], nosocomial pneumonia in ventilated patients [19], stroke-associated pneumonia [20], osteoporosis [21], obesity [22], metabolic syndrome [23], and chronic obstructive pulmonary disease [24].

Emerging evidence indicates that dysbiosis might contribute to carcinogenesis [25,26] through several mechanisms, including chronic inflammation, the production of genotoxins that can damage host DNA, and the disruption of host immune responses [26,27]. Moreover, specific taxa, such as *Helicobacter pylori*, were shown to express a protein called cytotoxin-associated gene A (CagA), which is secreted into the host cells and modulates beta-catenin signaling, which further up-regulates genes involved in proliferation, migration, and cell survival, thus promoting gastric cancer [28]. *Fusobacterium nucleatum* was linked to cancer development by modulating the NF-kB-driven proinflammatory response [28].

Although cancer is a multifactorial disease, shaped by genetic, environmental, and microbial factors, the oral microbiome’s role remains unexplored. Most microbiome-oncology research has centered on the gut, where experimental outcomes have associated specific taxa to therapy responses and toxicity modulations [27]. In contrast, the oral microbiome’s response to oncologic therapies remains poorly understood, despite increasing reports that chemotherapy and immunotherapy disrupt oral microbial composition, reduce diversity, and predispose to complications such as oral mucositis, candidiasis, and secondary infections [29,30]. These adverse effects not only impact patients’ quality of life but could also compromise treatment adherence and overall therapeutic outcomes [30].

Recent work suggests that cancer therapy not only decreases commensals (e.g., *Streptococcus*, *Actinomyces*, *Gemella*, *Granulicatella*, and *Veillonella*), but also favors enrichment of Gram-negative pathogens, including *Fusobacterium nucleatum*, *Prevotella oris*, *Escherichia*, *Shigella*, and *Megasphaera* [31,32,33]. Dysbiosis during treatment may exacerbate mucosal injury and systemic inflammation. Moreover, emerging data suggest that specific oral taxa could shape immune responses relevant to immunotherapy efficacy [34]. For example, *Fusobacterium nucleatum*, *Bacteroides fragilis*, and *Escherichia coli* could improve survival in patients receiving adoptive cell therapy, possibly by improving cytokine production and T-cell infiltration [35]. *Lactobacillus fermentum* has been shown to stimulate immune responses in patients treated with CpG-oligonucleotides that act as Toll-like receptor 9 agonists [36].

Advances in DNA sequencing, such as whole-metagenome shotgun sequencing and metatranscriptomics, have revolutionized microbiome research, leading to a more detailed, comprehensive view of microbial dynamics in response to cancer therapy, even for unculturable species [37,38]. In this respect, long-read nanopore sequencing stands out as a powerful tool for the real-time sensitive detection of microbial shifts and functional changes [39]. Unlike short-read sequencing approaches, nanopore technology provides long-read sequencing of the 16S rRNA gene, generating a rapid and comprehensive taxonomic identification (down to species level) of the microbial community within clinical samples. Therefore, even in low-biomass samples there is a significantly enhanced sensitivity and ability to analyze mixed bacterial populations [40,41]. In the context of oncology, there is a need for early detection of dysbiosis, monitoring of treatment-induced microbial shifts, and risk stratification for therapy-related complications [32]. By detecting microbial dysbiosis in near real-time [42], nanopore sequencing can support clinicians in personalized oral care interventions, potentially improving treatment tolerability and overall prognosis in cancer patients undergoing chemotherapy and/or immunotherapy. Thus, integrating microbiome surveillance into clinical practice may offer new avenues into precision oncology [42,43].

The aim of the present study was to characterize shifts in the oral microbiome induced by chemotherapy alone or in combination with immunotherapy, with the help of nanopore sequencing. By comparing the microbial outcomes of two treatment modalities, we wanted to provide a deeper insight into oral microbial changes and to explore their potential associations with oral mucositis and other treatment-associated complications in cancer patients. This could potentially lead to targeted oral care strategies, prophylactic interventions, or microbiome-supportive therapies in oncology patients.

## 2. Materials and Methods

### 2.1. Study Design and Sample Collection

**Figure 1:**
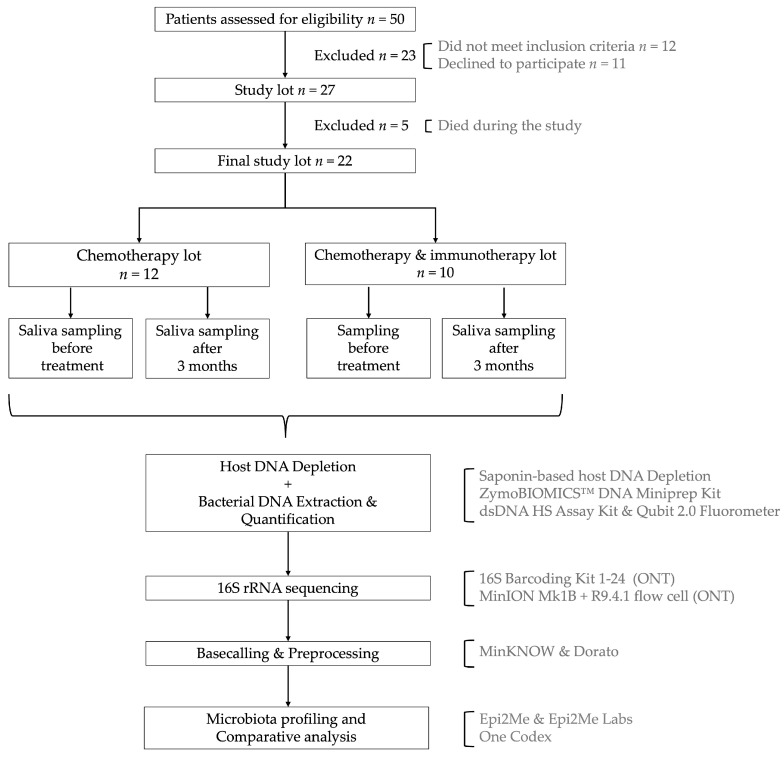
Graphical representation of the workflow.

We recruited 27 adult patients diagnosed with various types of solid tumors and oral mucosal lesions, enrolled between June 2023 and October 2023, at Oncocenter, Timisoara, Romania (Figure 1). All participants underwent either chemotherapy alone or in combination with immunotherapy. During the study, 5 patients passed away, leaving 22 subjects for continued analysis.

Saliva samples were collected using a DNA/RNA Shield SafeCollect Saliva Collection Kit (Zymo Research, Irvine, CA, USA) at two time points: before treatment initiation and three months post-treatment. All samples were anonymized and stored at −20 °C until further analysis. The study was conducted in accordance with the Declaration of Helsinki and received ethical approval from the Ethics Committee of “Victor Babes” University of Medicine and Pharmacy, Timisoara (Approval No. 76/16.11.2021, revised 26 May 2023). Written informed consent was obtained from all participants for sample collection, treatment administration, and subsequent investigations. Eligible participants were adult patients (≥18 years) diagnosed with histologically confirmed solid tumors and presenting with oral mucosal lesions, who were scheduled to undergo either chemotherapy or a combined chemo–immunotherapy treatment. Treatment was performed according to European Society for Medical Oncology (ESMO) guidelines (Supplementary Table S1). Exclusion criteria included antibiotic use within the past four weeks, known immunodeficiency disorders unrelated to cancer treatment, and lack of informed consent. Furthermore, none of the patients were under antibiotic therapy throughout the entire study. Their dietary regimen, hygiene, and chronic treatment were constant during the study.

### 2.2. Bacterial DNA Extraction and Purification

Due to the high concentration of host DNA in saliva samples [44], a host DNA depletion step was performed prior to bacterial DNA extraction using a saponin-based method [45], as described by Charalampous et al.

- Centrifugation: 2 mL of saliva preserved in DNA/RNA Shield (Zymo Research, Irvine, CA, US) was centrifuged at 10,000× *g* for 7 min.
- Supernatant Removal and Resuspension: The supernatant was discarded, and the pellet was resuspended in 250 µL phosphate-buffered saline (PBS) (Thermo Fisher, Waltham, MA, USA).
- Saponin Treatment: 200 µL of 5% saponin (Sigma-Aldrich, St. Louis, MO, USA) solution was added, followed by 10 min of incubation at room temperature.
- Cell Lysis:
  ○ 350 µL of nuclease free water was added and incubated for 30 s at room temperature.
  ○ 12 µL of 5M NaCl was introduced and the mixture was centrifuged at 6000× *g* for 5 min at room temperature.
- Heat-Labile Salt Active Nuclease (HL-SAN) Treatment: The pellet was resuspended in:
  ○ 100 µL PBS (Thermo Fisher, Waltham, MA, USA),
  ○ 100 µL HL-SAN Buffer (5.5 M NaCl + 100 mM MgCl_2_) (Sigma-Aldrich, St. Louis, MO, USA),
  ○ 10 µL HL-SAN DNase (Articzymes Technologies ASA, Tromsø, Norway),
  ○ Followed by incubation at 37 °C with shaking at 800 rpm for 15 min.
- Pellet Washing: After another centrifugation (6000× *g* for 3 min), the pellet was washed with 800 µL of PBS (Thermo Fisher, Waltham, MA, USA) and then 1000 µL of PBS (Thermo Fisher, Waltham, MA, USA), followed by a final centrifugation (6000× *g* for 3 min).
- Bacterial DNA Extraction and Purification: The final pellet was subjected to DNA extraction and purification using a ZymoBIOMICS^TM^ DNA Miniprep Kit (Zymo Research, Irvine, CA, USA), following the manufacturer’s protocol.

Purified DNA was quantified using a Qubit 2.0 Fluorometer (Invitrogen, Waltham, MA, USA) with a dsDNA High Sensitivity (dsDNA HS) Assay Kit (Thermo Fisher, Waltham, MA, USA). All DNA samples were stored at −20 °C until further processing.

### 2.3. Library Preparation for 16S rRNA Sequencing

For bacterial identification, 16S amplicon sequencing was performed using a 16S Barcoding Kit 1-24 (SQK-16S024, Oxford Nanopore Technologies (ONT), Oxford, UK) according to the manufacturer’s protocol. ONT sequencing was employed to enable full-length 16S rRNA gene sequencing, covering all variable regions, thereby enhancing taxonomic resolution compared to methods targeting single-variable regions [46].

Each sequencing run included 22 patient samples and 1 negative control. The prepared libraries were quantified using a Qubit 2.0 Fluorometer with a dsDNA HS Assay Kit (Thermo Fisher, Waltham, MA, USA). A DNA concentration of 50–100 ng was loaded onto a primed R9.4.1 flow cell (ONT, Oxford, UK), and sequencing was conducted using a MinION Mk1B instrument (ONT, Oxford, UK).

Raw data processing was performed using MinKNOW software v.23.11.2. Base calling, adapter trimming, demultiplexing, and quality control were carried out using Dorato v7.2.11 (ONT, Oxford, UK).

### 2.4. Sequencing Data Analysis

Microbiome data analysis was conducted using the Epi2me platform (v.2023.04.21-1804452), Epi2me Labs (v.1.5.0), and the One Codex (San Francisco, CA, USA) microbiome analysis platform [47]. Taxonomic classification was performed using targeted NCBI loci databases. To evaluate microbial diversity, we assessed alpha diversity (within-sample diversity), which reflects species richness and evenness in individual samples using Richness, Shannon, and Simpson indices. Beta diversity (between-sample diversity) was calculated using Jaccard dissimilarity and principal coordinate analysis (PCoA) to compare the compositional differences in microbiota across treatment groups and time points. Taxonomic composition was analyzed to determine relative abundances at the species level before and after treatment, allowing us to investigate the overall impact of chemotherapy alone and together with immunotherapy on oral microbiome.

### 2.5. Statistical Analysis

Statistical analysis was performed using Prism 10 for macOS (Version 10.4.1). Descriptive statistics were applied to summarize demographic and clinical data of the study participants. The Kolmogorov–Smirnov test was used to assess data distribution normality. A heteroscedastic Student’s *t*-test was conducted to compare normally distributed continuous variables. The Z-test was applied to analyze binary variable datasets. Statistical significance was set at *p* < 0.05, and all tests were two-tailed.

## 3. Results

The clinical characteristics of the study cohort are summarized in Table 1. A total of 22 cancer patients were enrolled, with a median age of 65 years (range: 42–75 years), and 59.09% of the participants were female (Supplementary Table S1).

Among the associated comorbidities, hypertension was the most prevalent, affecting 59.09% of the patients. Regarding cancer recurrence, 27.27% of the participants experienced a relapse, whilst 31.82% had a family history of cancer.

Following stratification (chemotherapy vs. combined chemo–immunotherapy), no statistically significant differences were observed between thetwo groups.

### 3.1. Sequencing Quality Control

In both sequencing rounds (Table 2), the average quality score was 10.05, exceeding the minimum quality threshold of 7, ensuring high confidence base calling. The average read length was approximately 1500 bases, closely matching the expected length of the 16S rRNA gene (~1600 bases).

### 3.2. Overall Microbiota Composition Before and After Treatment

In all patients, *Firmicutes* and *Proteobacteria* remained the dominant phylum both before and after treatment (Supplementary Figure S1); however, *Proteobacteria* showed a decreasing trend post-treatment. At the genus level, *Streptococcus*, *Veillonella*, and *Neisseria* were the most predominant before treatment. Post-treatment, *Streptococcus*, *Veillonella*, *Granulicatella*, and *Gemella* relative abundance increased, whereas *Neisseria* became less frequently detected (Supplementary Figure S2). This rise in facultative anaerobes and Gram-positive cocci may reflect a compensatory colonization dynamic.

α-diversity indices were significantly decreased in all patients following treatment. At the genus level, both the Shannon index (*p* = 0.0009) and the Simpson index (*p* = 0.0001) were significantly reduced three months after treatment compared to the pre-treatment baseline (Supplementary Figure S3). Similar trends were observed at the phylum and species levels (Supplementary Figures S4 and S5), indicating that oral microbiota diversity decreased upon therapy onset, consistent with dysbiosis typically associated with immunosuppression.

### 3.3. Microbiota Changes Following Chemotherapy Treatment

#### 3.3.1. Taxonomic Composition

**Figure 2:**
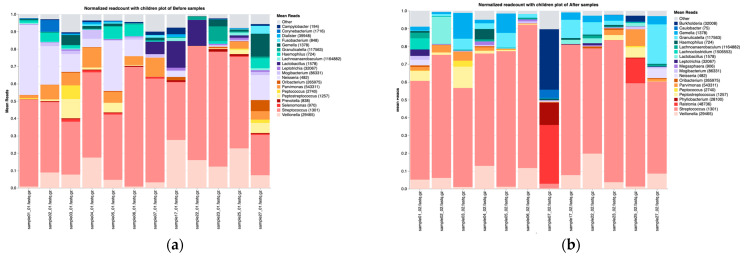
Taxonomic composition at the genus level before (**a**) and after (**b**) treatment with chemotherapy.

Upon stratification by type of therapy, patients receiving chemotherapy alone exhibited a consistent taxonomic distribution at the phylum level, with *Firmicutes* followed by *Proteobacteria* being the most dominant both before and after treatment (Supplementary Figure S6). At the genus level, the most abundant taxa before chemotherapy were *Streptococcus*, *Neisseria*, *Veillonella*, and *Lactobacillus*. Within three months of chemotherapy, the oral microbiota composition changed significantly and *Streptococcus*, *Gemella*, *Veillonella*, and *Granulicatella* became the most dominant genera. Overall, the post-treatment microbial profile is associated with an increased abundance of *Streptococcus*, *Gemella*, and *Granulicatella* and a decline in the abundance of *Neisseria* and *Veillonella* (Figure 2). These shifts could suggest an inflammatory or immune-modulatory treatment effect that favors more resilient Gram-positive cocci.

Notably, two samples (06 and 23) from patients who underwent chemotherapy exhibited a high abundance of specific taxa, with *Streptococcus salivarius*, followed by *Streptococcus parasanguinis* and *Streptococcus salivarius* (at a moderate-abundance level), being the predominant species before and after treatment.

Low-abundance taxa including Parvimonas micra, Streptococcus mitis, Gemella sanguinis, Peptostreptococcus stomatis, and Veillonella parvula were consistently observed.

In samples no. 06, 13, 04, 05, and 07 we identified several rare bacterial species, including *Cardiobacterium hominis*, *Porphyromonas pasteri*, *Aggregatibacter segnis*, *Haemophilus sputorum*, and *Sphingomonas paucimobilis* (Supplementary Table S2). These low-abundance, yet potentially pathogenic taxa, may play a role in treatment-associated complications, but require a deeper investigation.

#### 3.3.2. Alpha Diversity

**Figure 3:**
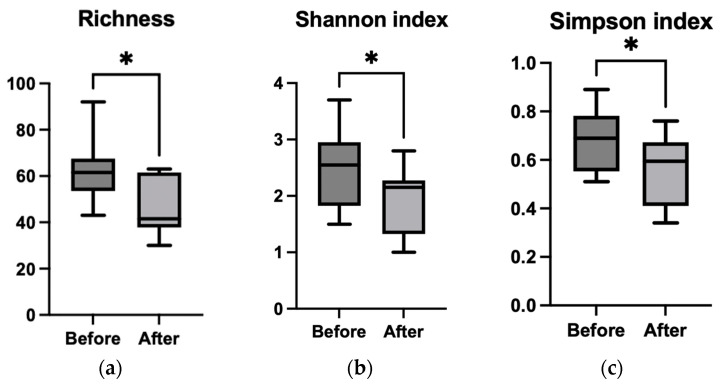
Comparison of the alpha diversity indices (Richness—(**a**); Shannon index—(**b**); Simpson index—(**c**)) for genus, before and after chemotherapy treatment using paired Student’s *t* test; * *p* < 0.05.

Alpha diversity metrics revealed statistically significant differences between the pre- and post-treatment groups, with Richness (*p* = 0.021), the Shannon index (*p* = 0.036), and the Simpson index (*p* = 0.019) being significantly lower in patients after chemotherapy (Figure 3a–c). Similar reductions in diversity were also observed at the phylum and species levels (Supplementary Figures S7 and S8), indicating a notable loss of microbial diversity following treatment, which may predispose patients to mucosal damage.

#### 3.3.3. Beta Diversity

**Figure 4:**
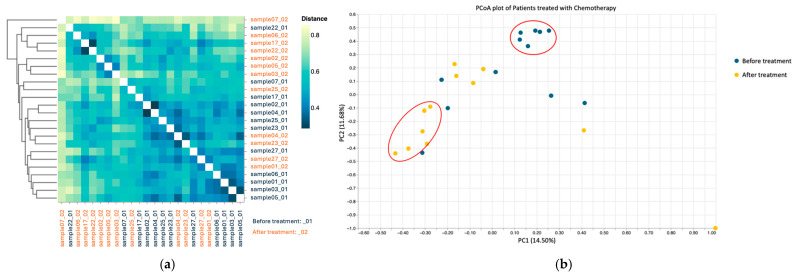
Beta diversity using Jaccard distance for patients treated with chemotherapy (**a**); PCoA based on Jaccard dissimilarity for chemotherapy group (**b**). Patients before treatment are represented in dark blue, and patients after treatment in orange.

Both beta diversity assessments by Jaccard distance matrices (Figure 4a) and PCoA (Figure 4b) revealed substantial dissimilarity between pre- and post-chemotherapy samples, with pre-treatment samples clustering separately from the post-treatment ones, indicating cohesive microbial community behavior.

### 3.4. Microbiota Changes in Patients Treated with Combined Chemo–Immunotherapy

#### 3.4.1. Taxonomic Composition

**Figure 5:**
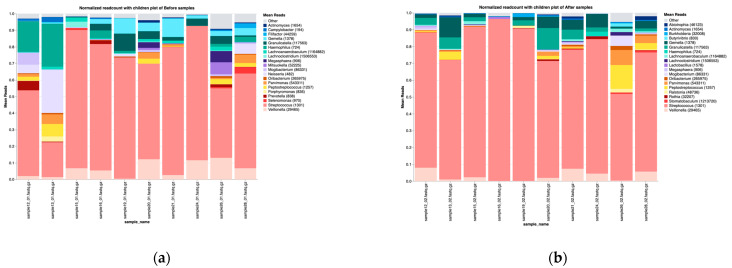
Taxa–genus before (**a**) and after (**b**) treatment with chemotherapy combined with immunotherapy.

In patients receiving combined chemo–immunotherapy, the most abundant phylum was *Firmicutes*, followed by *Proteobacteria* (Supplementary Figure S9). At the genus level, the predominant taxa before treatment included *Streptococcus*, followed by *Veillonella*, *Haemophilus*, and *Neisseria*. Post-treatment, *Streptococcus* relative abundance increased, *Veillonella* showed a slight decline, and *Haemophilus* and *Neisseria* experienced a substantial decrease, whilst *Granulicatella* and *Gemella* remained relatively stable (Figure 5).

Among patients treated with combined chemo–immunotherapy, only three samples exhibited a high abundance of specific taxa. In sample 12, *Streptococcus parasanguinis* was highly abundant before treatment but decreased to moderate abundance post-treatment; whereas in samples 15 and 24 *Streptococcus salivarius* was highly abundant before treatment and this trend persisted only in Sample 15 post-treatment.

In most samples, *Streptococcus parasanguinis* and *Streptococcus salivarius* were the most frequently detected species at moderate abundance, followed by *Streptococcus infantis*, *Neisseria mucosa*, and *Streptococcus gordonii*.

*Megasphaera micronuciformis* was identified once in the moderate-abundance category and appeared twice in the low-abundance category.

The most frequently encountered low-abundance taxa included *Gemella sanguinis, Streptococcus mitis*, *Streptococcus sanguinis*, *Streptococcus infantis*, *Streptococcus anginosus*, *Peptostreptococcus stomatis*, and *Parvimonas micra.*

Additionally, the following rare taxa were detected in low abundance: *Shuttleworthia satelles*, *Filifactor alocis*, *Streptococcus australis*, *Campylobacter showae*, and *Streptococcus sinensis* (Supplementary Table S2).

#### 3.4.2. Alpha Diversity

**Figure 6:**
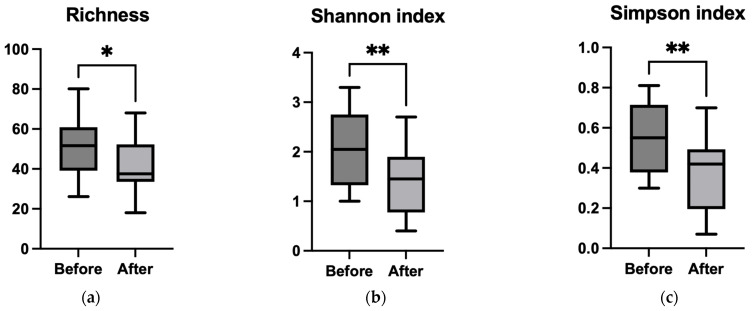
Comparison of the alpha diversity indices (Richness—(**a**); Shannon index—(**b**); Simpson index—(**c**)) for genus before and after chemotherapy combined with immunotherapy treatment using a paired Student’s *t* test; * *p* < 0.05;** *p* < 0.001.

In terms of Richness (Figure 6a), we noticed a statistically significant (*p* = 0.046) decrease, which was less intense for the chemotherapy group. Interestingly, both the Shannon (*p* = 0.0097) and Simpson (*p* = 0.0076) alpha diversity indices showed a significant decreasing trend in patients following combined chemo–immunotherapy only at the genus level (Figure 6b,c). The same pattern was identified in phylum and species, although the differences were not statistically significant (Supplementary Figures S10 and S11).

#### 3.4.3. Beta Diversity

**Figure 7:**
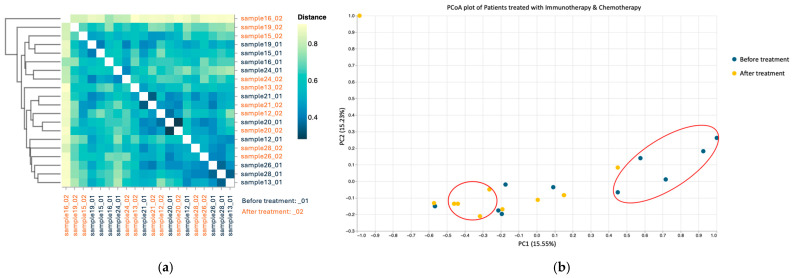
Beta diversity using Jaccard distance for patients treated with chemotherapy combined with immunotherapy; dark blue—patients before treatment and orange—patients after treatment (**a**); PCoA based on Jaccard dissimilarity for chemotherapy combined with immunotherapy group (**b**).

Jaccard-based beta diversity analysis (Figure 7a) demonstrated a shift in community composition between pre- and post-treatment states. However, the PCoA plots (Figure 7b) showed less distinct clustering than in the chemotherapy treated group, suggesting a more heterogenous microbial response in patients receiving combined therapy. This variability might reflect differential immunomodulatory effects or baseline microbiome differences influencing treatment outcomes.

### 3.5. Comparison Between Group Treated with Chemotherapy and Group Treated with Combined Therapy

**Figure 8:**
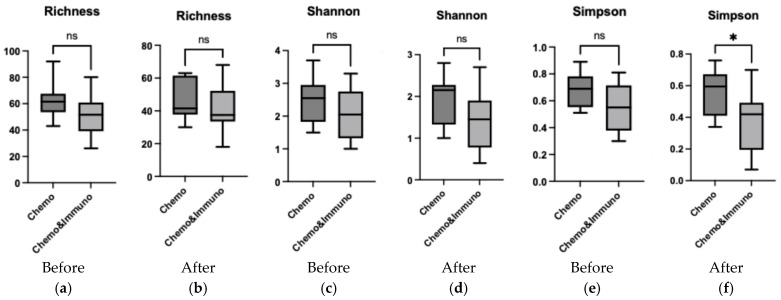
Comparison of the alpha diversity indices (Richness—(**a**,**b**); Shannon index—(**c**,**d**); Simpson index—(**e**,**f**)) for genus between patients treated with chemotherapy (Chemo) and patients treated with chemotherapy and immunotherapy (Chemo&Immuno) using Student’s *t* test; * *p* < 0.05, ns, not significant.

Alpha diversity analysis (Figure 8) revealed that prior to treatment, there were no significant differences between the chemotherapy-only and chemo–immunotherapy groups across Richness, Shannon, and Simpson indices (all *p* > 0.05). Post-treatment, although Richness and Shannon diversity remained comparable between groups, a significant reduction in Simpson diversity was observed in the chemo–immunotherapy cohort compared to chemotherapy alone (*p* = 0.025), indicating a loss of microbial evenness following combined therapy.

**Figure 9:**
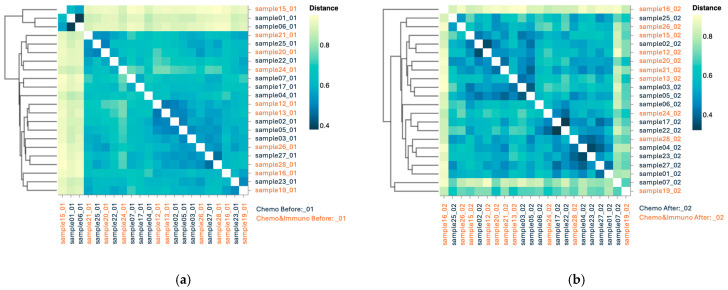
Beta diversity using Jaccard distance for patients before treatment (**a**) and patients after treatment (**b**); Chemotherapy patients (Chemo) are indicated in dark blue and combined therapy (Chemo&Immuno) is indicated in orange.

**Figure 10:**
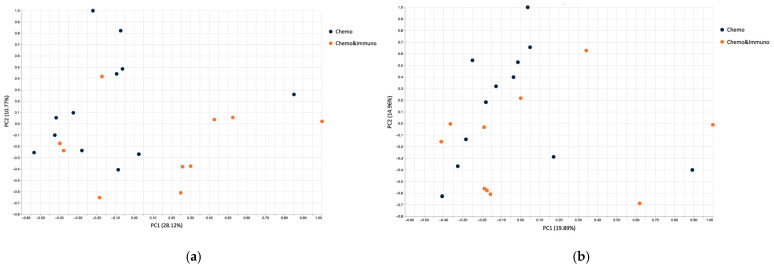
PCoA based on Jaccard dissimilarity for patients treated with chemotherapy (dark blue dots) and patients treated with chemotherapy and immunotherapy (orange dots) before treatment (**a**) and after treatment (**b**).

Beta diversity metrics (Figure 9) indicated moderate variability between individuals before treatment, with greater post-treatment dispersion in the chemo–immunotherapy group. PCoA (Figure 10) further illustrated this, showing tighter clustering in the chemotherapy group and broader scatter in the combined therapy group, implying more pronounced and variable microbiome disruption.

## 4. Discussion

The oral microbiome plays a vital role in maintaining mucosal immunity, preventing pathogen colonization, and modulating systemic immune responses [34]. While most microbiome research in oncology has focused on the gut, recent studies suggest that the oral microbiota may also influence treatment responses, toxicity profiles, and possibly even disease progression in cancer patients [48,49,50,51]. 

In this study, we employed nanopore sequencing to explore shifts in the oral microbiome among patients undergoing chemotherapy alone or in combination with immunotherapy. Our findings show that these treatments, particularly in patients receiving chemotherapy alone, are associated with microbial alterations indicative of dysbiosis, a state linked with mucosal inflammation, impaired barrier function, and an increased risk of opportunistic infections [52,53,54]. These changes are consistent with those previously described in the gut and oral microbiome during oncologic therapies and have been associated with adverse effects such as oral mucositis and candidiasis, both of which compromise patient quality of life and adherence to treatment [34,53].

Beta diversity analysis confirmed substantial compositional changes following therapy. Samples from chemotherapy-only patients clustered more tightly post-treatment, indicating a more uniform, potentially deleterious microbial shift. In contrast, the microbiota profiles from patients receiving combined chemotherapy and immunotherapy were more heterogenous. This suggests that the addition of immunotherapy might partially preserve microbial variability or affect host–microbiome interactions in a more individualized manner. Whether this heterogeneity translates to better clinical outcomes or reduced toxicity remains to be elucidated.

At the phylum level, in both treatment groups *Firmicutes* and *Proteobacteria* remained dominant. At the genus level, we found that *Streptococcus* abundance increased in both treatment groups. While *Streptococcus* includes common commensal species, it also comprises opportunistic pathogens that can exacerbate inflammation and mucosal damage under immunosuppressive conditions [55].

Importantly, *Veillonella* and *Neisseria*, both of which contribute to oral and systemic homeostasis [4], declined post-treatment. While *Veillonella* is generally a commensal bacterium, fluctuations in its abundance have been associated with periodontitis, endocarditis, and sepsis, particularly in immunocompromised patients [56]. *Veillonella* depletion from oral microbiota was linked to mucositis-associated dysbiosis, a pattern also observed in a study of patients who developed mucositis [31,57]. Non-pathogenic *Neisseria* species contribute to oral health by metabolizing nitrate to nitrite, a compound with antimicrobial properties [58]. A decrease in *Neisseria* abundance, as observed in both treatment groups, could impact nitric oxide production and mucosal barrier integrity, potentially increasing not only oral disease susceptibility [58,59] but also systemic effects, including altered blood pressure regulation and elevated inflammatory responses [60]. These findings highlight the potential need for strategies aimed at preserving or restoring nitrate-reducing microbiota during cancer therapy to maintain both oral and systemic health.

Interestingly, the chemotherapy group exhibited an increase in *Gemella* and *Granulicatella*, while in the combined group their levels remained stable. These two genera are typically considered commensal but capable of opportunistic pathogenicity, particularly in immunocompromised hosts [61]. Their relative stability in the chemo–immunotherapy group suggests that immunotherapy may exert a stabilizing effect on the microbial ecosystem or promote better mucosal immune surveillance. Moreover, *Gemella* species have been reported to be enriched in patients with oral squamous-cell carcinoma, raising questions about their role in cancer-associated microbial shifts [50].

These findings align with emerging evidence that cancer therapies, especially immunotherapies, interact with host microbiota in complex ways. While the gut microbiome has been extensively studied for its impact on immunotherapy response, research on the oral microbiome’s role remains limited [62]. Some oral taxa, such as *Fusobacterium nucleatum*, have been associated with poor prognostic outcomes in several cancers, highlighting the potential for microbiota-based predictive biomarkers [63]. Our observation that microbial dysbiosis was more pronounced in the chemotherapy-only group may point toward the protective or modulatory role of immunotherapy in preserving oral microbial balance, though further mechanistic studies are needed.

The clinical implications of oral dysbiosis in oncology are increasingly recognized. Identifying microbial patterns predictive of complications like mucositis or a poor immunotherapy response could pave the way for microbiota-guided supportive strategies [31,64]. Furthermore, in the context of immunotherapy, the microbiome may influence treatment efficacy through immune modulation, as observed in studies of the gut microbiota [65]. A similar interaction could occur at the oral level, where microbial composition might shape local or systemic immune responses [66]. These insights underline the need for future research to explore therapeutic strategies—such as microbiome-supportive interventions, probiotics/prebiotics administration, or tailored oral hygiene protocols—that could preserve eubiosis and potentially enhance patient outcomes during oncological treatment by managing therapy-related toxicities and improving treatment tolerance [67].

In spite of the fact that our study is a pilot study with limitations in addressing all potential confounding factors, it provides an initial framework for future research. The main disadvantages are the small cohort size, the lack of extended longitudinal data, and the reliance on 16S sequencing without functional inference, which limits the general applicability of our findings. Even though we excluded recent (4 weeks) antibiotic users, unrecorded antimicrobial exposure and oral-hygiene differences may still confound the findings. However, this pilot investigation offers valuable insights into the modifications of the oral microbiota during cancer treatment, laying the foundation for more extensive studies that can further explore these microbiome changes across different phases of therapy. Moreover, it could reveal opportunities for targeted interventions to restore microbial balance during cancer therapy and avoid resulting complications.

## 5. Conclusions

Although limited by its small size, requiring validation in a larger, adequately powered cohort stratified by therapy modality and tumor type, this study revealed significant changes in the oral microbiota of cancer patients undergoing chemotherapy or combined chemo–immunotherapy. We show that chemotherapy alone is associated with an increased relative abundance of *Streptococcus*, *Gemella*, and *Granulicatella*, alongside a reduction in *Neisseria* and *Veillonella*. Patients receiving both chemotherapy and immunotherapy exhibited a more heterogeneous microbiota response with a notable increase in *Streptococcus*, a slight decline in *Veillonella*, and a pronounced decrease in *Haemophilus* and *Neisseria*, whilst *Granulicatella* and *Gemella* remained relatively stable. A marked reduction in microbial richness and diversity post-treatment highlights the impact of these therapies on the oral microbiome and their role in complications such as oral mucositis. Our findings emphasize the importance of incorporating microbiome monitoring into oncologic care to better understand treatment-induced microbial shifts. Future studies should validate these results in larger, stratified cohorts and explore targeted interventions to preserve oral eubiosis, improving treatment tolerance and reducing the burden of therapy-related toxicities.

## Figures and Tables

**Table 1:** Demographic and clinical characteristics of the patients.

	All *n* = 22	Chemotherapy *n* = 12	Chemotherapy and Immunotherapy *n* = 10	*p*
Age ± SD	62.86 ± 9.57	64.33 ± 7.28	61.1 ± 11.95	0.467 *
Male/Female	9/13	3/9	6/4	0.097 **
Hypertension	13 (59.09)	7 (58.33)	6 (60)	0.936 **
Diabetes mellitus	6 (27.27)	3 (25)	3 (30)	0.795 **
Thyroid affection	3 (13.64)	2 (16.64)	1 (10)	0.653 **
Smoking	6 (27.27)	3 (25)	3 (30)	0.795 **
Allergies	3 (13.64)	1 (8.33)	2 (20)	0.430 **
Cancer in family history	7 (31.82)	4 (33.33)	3 (30)	0.865 **
Cancer relapse	6 (27.27)	3 (25)	3 (30)	0.795 **
Removable dental prosthesis	10 (45.45)	5 (41.67)	5 (50)	0.697 **
Oral thrush	9 (40.91)	7 (58.33)	2 (20)	0.069 **
Mucositis	5 (22.73)	2 (16.64)	3 (30)	0.459 **
Gingivitis	8 (36.36)	3 (25)	5(50)	0.023 **
**Cancer Type**				
Breast cancer	8 (36.36)	6 (50)	2 (20)	0.144 **
Liposarcoma	1 (4.54)	1 (8.33)	0 (0)	0.352 **
Stomach cancer	1 (4.54)	1 (8.33)	0 (0)	0.352 **
Melanoma	2 (9.09)	0 (0)	2 (20)	0.105 **
Lung cancer	5 (22.73)	2 (16.64)	3 (30)	0.459 **
Cervical cancer	2 (9.09)	0 (0)	2 (20)	0.105 **
Gallbladder cancer	1 (4.54)	1 (8.33)	0 (0)	0.352 **
Rectal cancer	2 (9.09)	1 (8.33)	1 (10)	0.889 **

SD—standard deviation; * unpaired heteroscedastic Student *t*-test; ** two-tailed Z-test.

**Table 2:** Quality control parameters.

QC	Seq 1	Seq 2
Reads Analyzed	1,388,589	2,199,826
Total Yield	2.1 Gbases	3.4 Gbases
Avg Quality Score	10.19	10.04
Avg Sequence Length	1542	1539

## Data Availability

The original contributions presented in this study are included in the article/Supplementary Material.

## Supplementary Materials

The following supporting information can be downloaded at: https://www.mdpi.com/article/10.3390/microorganisms13071565/s1, Figure S1. Taxonomic composition at the phylum level before (a) and after (b) treatment in all patients; Figure S2. Taxonomic composition at the genus level before (a) and after (b) treatment in all patients; Figure S3. Overall comparison of the alpha diversity indexes (Shannon Index—(a); Simpson Index—(b)) for genus, before and after treatment using paired Student’s T test; Figure S4. Overall comparison of the alpha diversity indexes (Shannon Index—(a); Simpson Index—(b)) for phylum, before and after treatment using paired Student’s T test; Figure S5. Overall comparison of the alpha diversity indexes (Shannon Index—(a); Simpson Index—(b)) for species, before and after treatment using paired Student’s T test; Figure S6. Taxonomic composition at the phylum level before (a) and after (b) chemotherapy treatment; Figure S7. Comparison of the alpha diversity indexes (Shannon Index—(a); Simpson Index—(b)) for phylum, before and after chemotherapy treatment using paired Student’s T test; Figure S8. Comparison of the alpha diversity indexes (Shannon Index—(a); Simpson Index—(b)) for species, before and after chemotherapy treatment using paired Student’s T test; Figure S9. Taxonomic composition at the phylum level before (a) and after (b) combined chemotherapy and immunotherapy treatment; Figure S10. Comparison of the alpha diversity indexes (Shannon Index—(a); Simpson Index—(b)) for phylum, before and after combined chemotherapy and immunotherapy treatment using paired Student’s T test; Figure S11. Comparison of the alpha diversity indexes (Shannon Index—(a); Simpson Index—(b)) for species, before and after combined chemotherapy and immunotherapy treatment using paired Student’s T test; Table S1 Patients clinical data; Table S2 Bacterial abundance.
