# Supplementary material for: Impact of Chemotherapy Alone and in Combination with Immunotherapy on Oral Microbiota in Cancer Patients—A Pilot Study
Source: Microorganisms. 2025 Jul 3;13(7):1565. doi: 10.3390/microorganisms13071565 (PMC12299619; doi:10.3390/microorganisms13071565)
Supplement: Supplementary file 1 [file microorganisms-13-01565-s001.zip › Supplementary Figures.pdf]

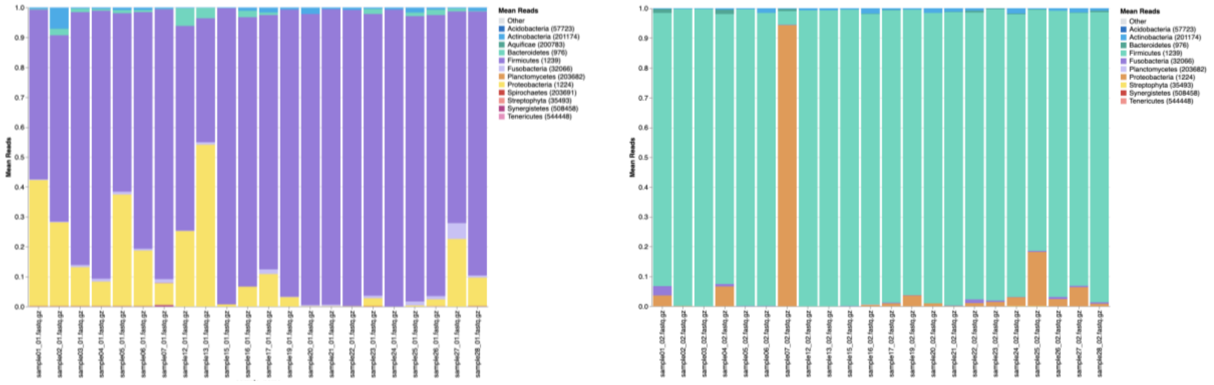

(a) (b)

Figure 1. Taxonomic composition at the phylum level before (a) and after (b) treatment in all patients

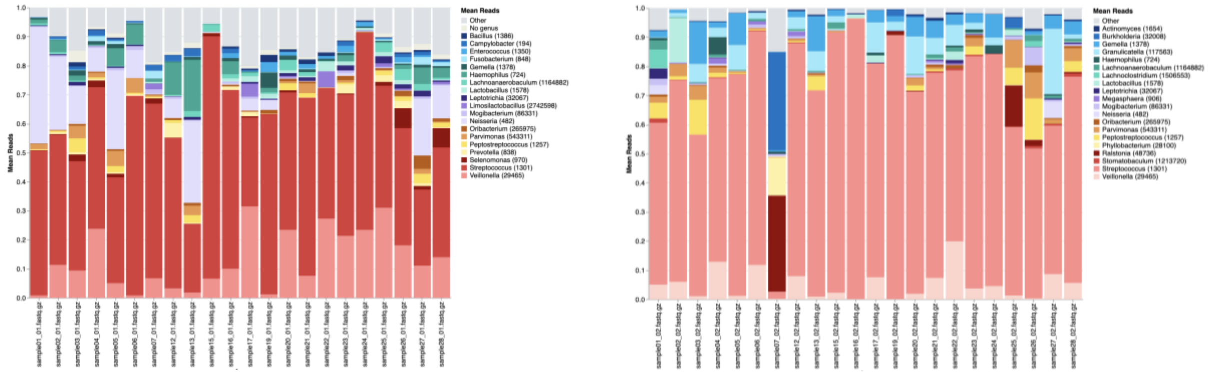

(a) (b)

Figure 2. Taxonomic composition at the genus level before (a) and after (b) treatment in all patients

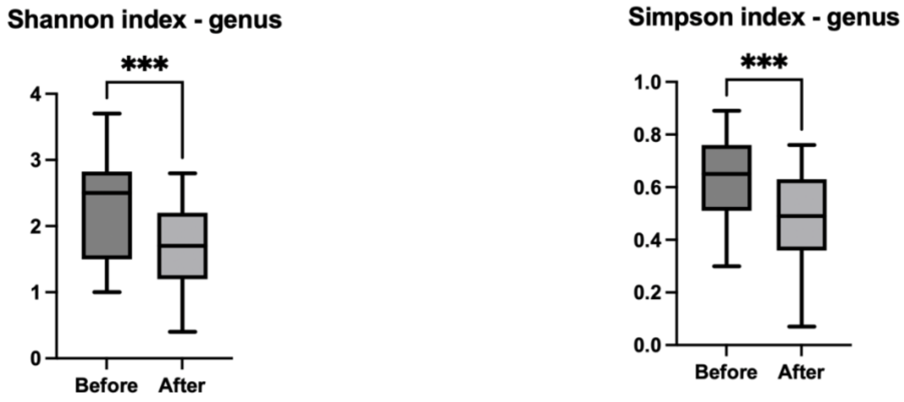

(a) (b)

Figure 3. Overall comparison of the alpha diversity indexes (Shannon Index – (a); Simpson Index – (b)) for genus, before and after treatment using paired Student's T test

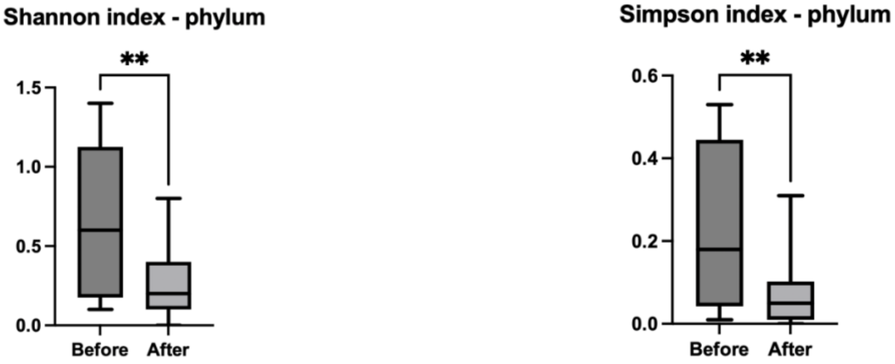

(a) (b)

Figure 4. Overall comparison of the alpha diversity indexes (Shannon Index – (a); Simpson Index – (b)) for phylum, before and after treatment using paired Student's T test

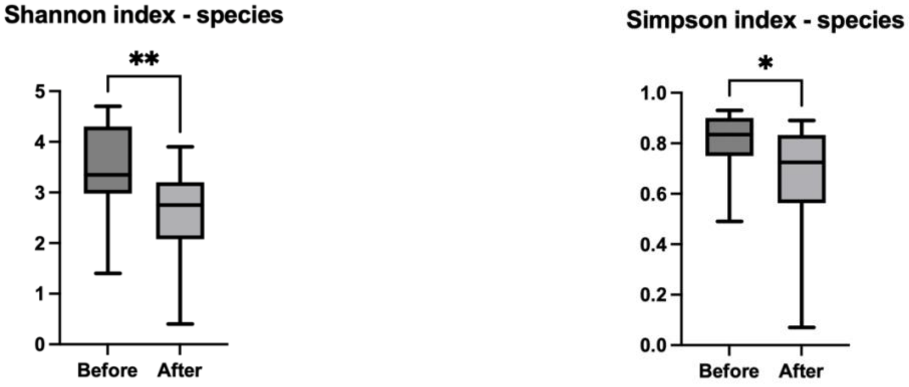

(a) (b)

Figure 5. Overall comparison of the alpha diversity indexes (Shannon Index – (a); Simpson Index – (b)) for species, before and after treatment using paired Student's T test

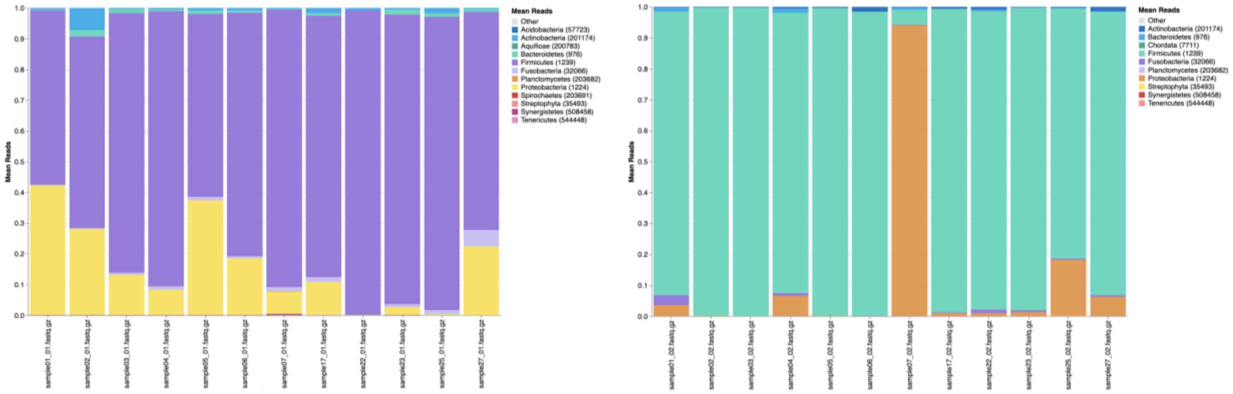

(a) (b)

Figure 6. Taxonomic composition at the phylum level before (a) and after (b) chemotherapy treatment

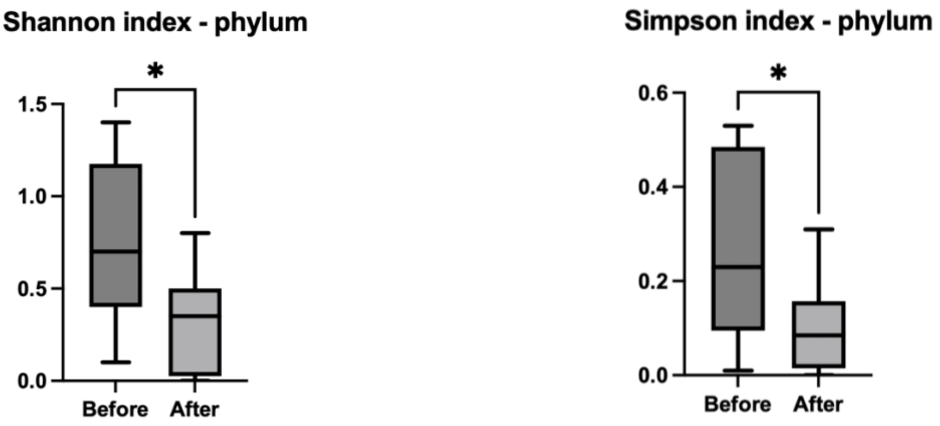

(a) (b)

Figure 7. Comparison of the alpha diversity indexes (Shannon Index – (a); Simpson Index – (b)) for phylum, before and after chemotherapy treatment using paired Student's T test

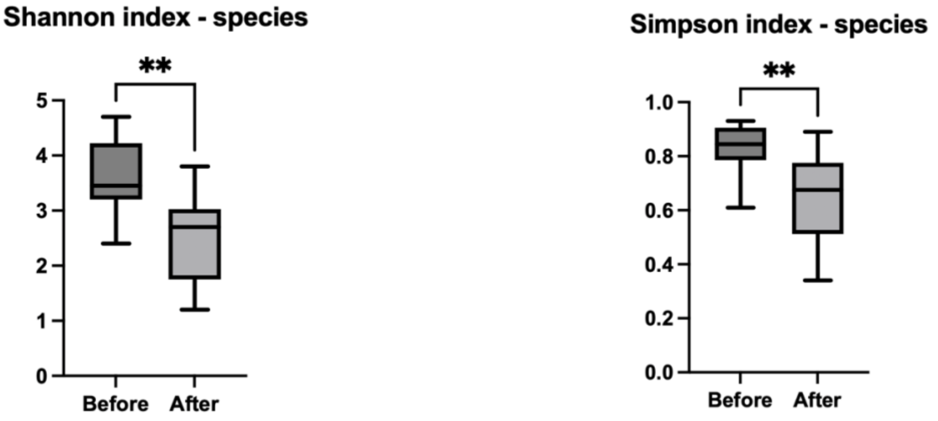

(a) (b)

Figure 8. Comparison of the alpha diversity indexes (Shannon Index – (a); Simpson Index – (b)) for species, before and after chemotherapy treatment using paired Student's T test

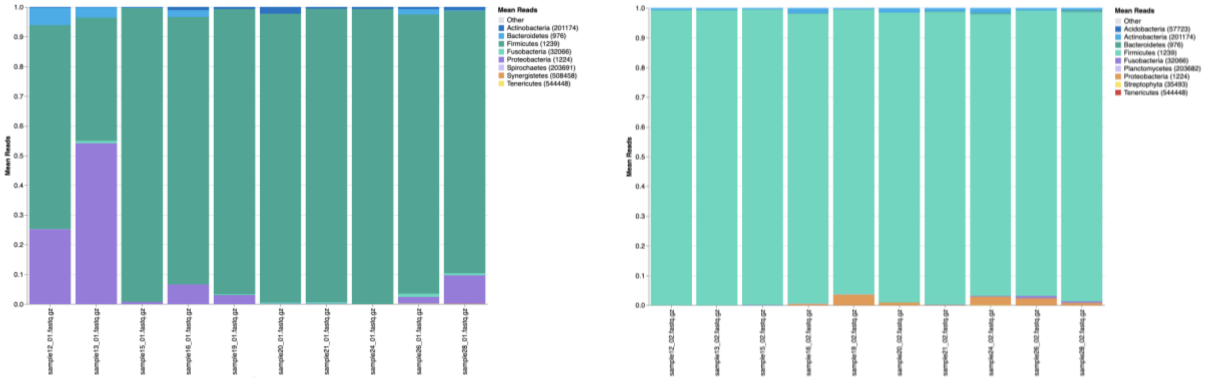

(a) (b)

Figure 9. Taxonomic composition at the phylum level before (a) and after (b) combined chemotherapy and immunotherapy treatment

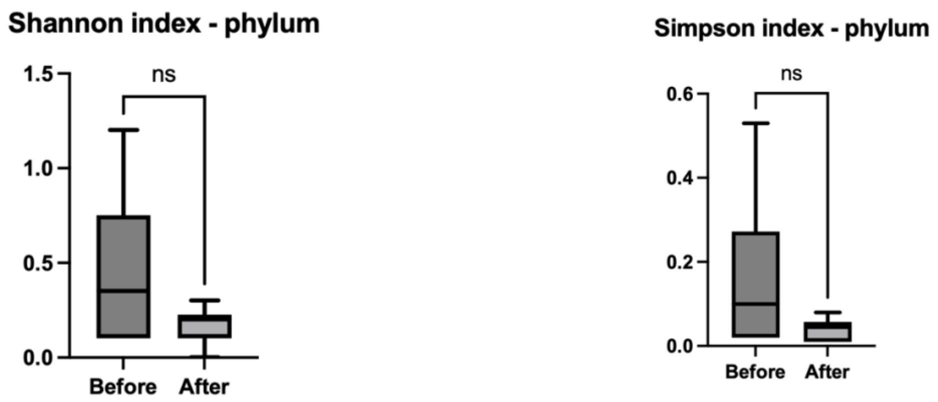

(a) (b)

Figure 10. Comparison of the alpha diversity indexes (Shannon Index – (a); Simpson Index – (b)) for phylum, before and after combined chemotherapy and immunotherapy treatment using paired Student's T test

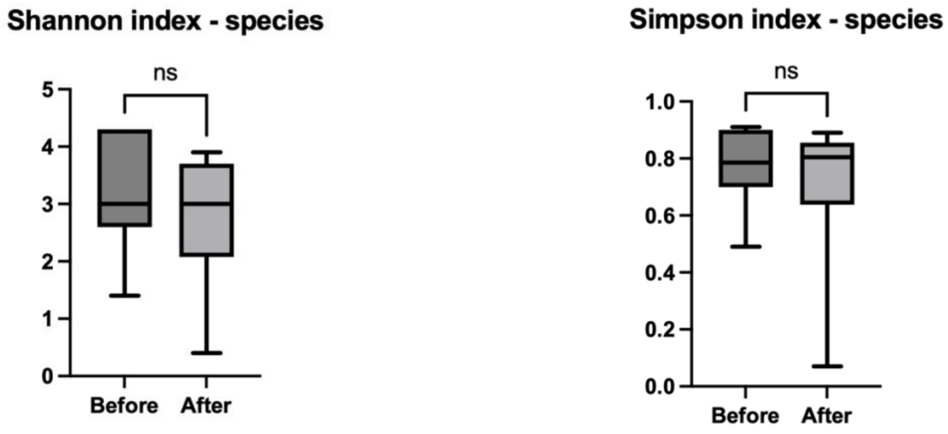

(a) (b)

Figure 11. Comparison of the alpha diversity indexes (Shannon Index – (a); Simpson Index – (b)) for species, before and after combined chemotherapy and immunotherapy treatment using paired Student's T test
